# Establishing mouse and human oral esophageal organoids to investigate the tumor immune response

**DOI:** 10.1242/dmm.050319

**Published:** 2024-01-23

**Authors:** Yuan Jiang, Hua Zhao, Shuai Kong, Dan Zhou, Jinxiu Dong, Yulan Cheng, Shuo Zhang, Fei Wang, Andrew Kalra, Nina Yang, Dan-Dan Wei, Jian Chen, Yuan-Wei Zhang, De-Chen Lin, Stephen J. Meltzer, Yan-Yi Jiang

**Affiliations:** ^1^ Institute of Health and Medical Technology, Hefei Institutes of Physical Science, Chinese Academy of Sciences, Hefei 230031, China; ^2^ University of Science and Technology of China, Hefei 230026, China; ^3^ Center for Craniofacial Molecular Biology, Herman Ostrow School of Dentistry, and Norris Comprehensive Cancer Center, University of Southern California, Los Angeles, CA 90033, USA; ^4^ Division of Gastroenterology and Hepatology, Department of Medicine and Oncology, Sidney Kimmel Comprehensive Cancer Center, Johns Hopkins University School of Medicine, Baltimore, MD 21287, USA; ^5^ Institutes of Physical Science and Technology, Anhui University, Hefei 230601, China; ^6^ Hefei Cancer Hospital, Chinese Academy of Sciences, Hefei 230031, China

**Keywords:** Organoids, Immune response, Oral and esophageal cancer

## Abstract

Organoid culture systems are very powerful models that recapitulate *in vivo* organ development and disease pathogenesis, offering great promise in basic research, drug screening and precision medicine. However, the application of organoids derived from patients with cancer to immunotherapeutic research is a relatively untapped area. Esophageal cancer is one of the most lethal malignancies worldwide, including two major pathological subtypes: esophageal squamous cell carcinoma (ESCC) and esophageal adenocarcinoma. ESCC shares many biological and genomic features with oral squamous cell cancers. Herein, we provide a versatile protocol for the establishment and maintenance of oral and esophageal organoid cultures derived from both murine and human samples. We describe culture conditions for organoids derived from normal tongue, esophagus and gastroesophageal junction, esophageal cancer and Barrett's esophagus. In addition, we establish an *ex vivo* model by co-culturing patient tumor-derived organoids and autologous CD8^+^ T lymphocytes to assess CD8^+^ T cell-mediated tumor killing. Our protocol can also be modified for organoid establishment from other squamous epithelia and carcinomas. The co-culture model can serve as a template for studies of other tumor-immune cell interactions and the efficacy of immune checkpoint blockade therapy.

## Teaser

**Summary:** We describe optimized protocols to generate organoid cultures from mouse esophageal and tongue epithelia and patient-derived esophageal samples. We also establish a co-culture model to assess CD8^+^ T cell-mediated tumor killing in patient samples.

## INTRODUCTION

Organoids are three-dimensional (3D) cell cultures based on the principles of stem cell differentiation. Compared with traditional two-dimensional (2D) cell cultures, organoids exhibit complex structures and functions that more closely recapitulate the tissues and organs from which they are derived, providing a superior model for studies of development and disease pathogenesis (Drost and Clevers, 2018; Lancaster and Knoblich, 2014; Tuveson and Clevers, 2019). In addition, relative to animal models, it is more convenient and feasible to establish organoids, while avoiding the potential risk of introducing divergent pathological or anatomical features unique to certain animals (Lancaster and Knoblich, 2014). Finally, in cancer research, the establishment of patient-derived organoid biobanks will facilitate screening of anti-cancer drugs, while contributing to the development of personalized (precision) therapeutics (Drost and Clevers, 2018; Pauli et al., 2017; Rossi et al., 2018; Tuveson and Clevers, 2019).

Esophageal cancer (EC) is one of the most common and lethal malignancies worldwide, causing over 600,000 deaths annually (Bray et al., 2018; Sung et al., 2021). EC comprises two major histological subtypes: esophageal squamous cell carcinoma (ESCC) and esophageal adenocarcinoma (EAC); ESCC and EAC display substantial differences biologically, geographically and etiologically (Arnold et al., 2020). EAC represents the primary EC subtype in North American/European populations (Arnold et al., 2020; Coleman et al., 2018), and is known to arise from premalignant Barrett's esophagus (BE), an intestinal metaplastic lesion (Fitzgerald, 2006; Rustgi and El-Serag, 2014). ESCC predominates in Eastern Asian and African regions, accounting for more than 85% of all EC worldwide (Arnold et al., 2020). The etiology and biology of ESCC strongly resemble those of human papillomavirus-negative oral cancers (OCs), which originate from oral squamous epithelial cells. One of the long-lasting challenges in OC and EC research is a lack of realistic disease models. In this context, organoid technologies are beginning to overcome this road block. Indeed, we and others have developed oral and esophageal organoid cultures to model normal oral and esophageal development (Kasagi et al., 2018; Sato et al., 2011; Trisno et al., 2018; Zhang et al., 2018), as well as oral and esophageal carcinogenesis (Driehuis et al., 2019; Driehuis et al., 2020; Kijima et al., 2019; Li et al., 2018; Liu et al., 2018; Whelan et al., 2018; Zhao et al., 2022). Notwithstanding this progress in OC and EC modeling, relative to that for other common cancer types, including colon cancer (Pauli et al., 2017; van de Wetering et al., 2015), gastrointestinal cancers (Sato et al., 2011; Vlachogiannis et al., 2018), pancreatic cancer (Boj et al., 2015; Broutier et al., 2016), breast cancer (Dekkers et al., 2021; Sachs et al., 2018) and bladder cancer (Lee et al., 2018), organoid modeling of OC and EC requires further development, optimization and validation. Moreover, research on OC and EC organoids will benefit from expanded applications, including studies of the tumor immune response and drug resistance, which have not been explored in detail in these organs.

In recent years, immunotherapy, such as immune checkpoint blockade (ICB), has demonstrated impressive clinical activities and has provided survival benefits for patients with many types of cancer (Hellmann et al., 2019; Hudson and Wieland, 2023; Wolchok et al., 2017; Xin Yu et al., 2020), including EC (Doki et al., 2022; Janjigian et al., 2021; Lu et al., 2022; Sun et al., 2021). Among the various ICB therapeutic strategies, T cells, especially CD8^+^ T cells, serve as the primary targets for mediating anti-tumor immunity owing to their direct ability to kill tumor cells (Hudson and Wieland, 2023; Robert, 2020). However, it is difficult to predict which individuals will respond to ICB therapy or to compare the effects of different immunotherapeutic strategies prior to treatment. Therefore, establishment of an *ex vivo* co-culture system with patient-derived tumor organoids and autologous cytotoxic T cells will provide insights into tumor intrinsic immune responses. More importantly, this approach has the potential to better inform personalized immunotherapy (Bar-Ephraim et al., 2020).

### Overview of the protocol

**Fig. 1.**
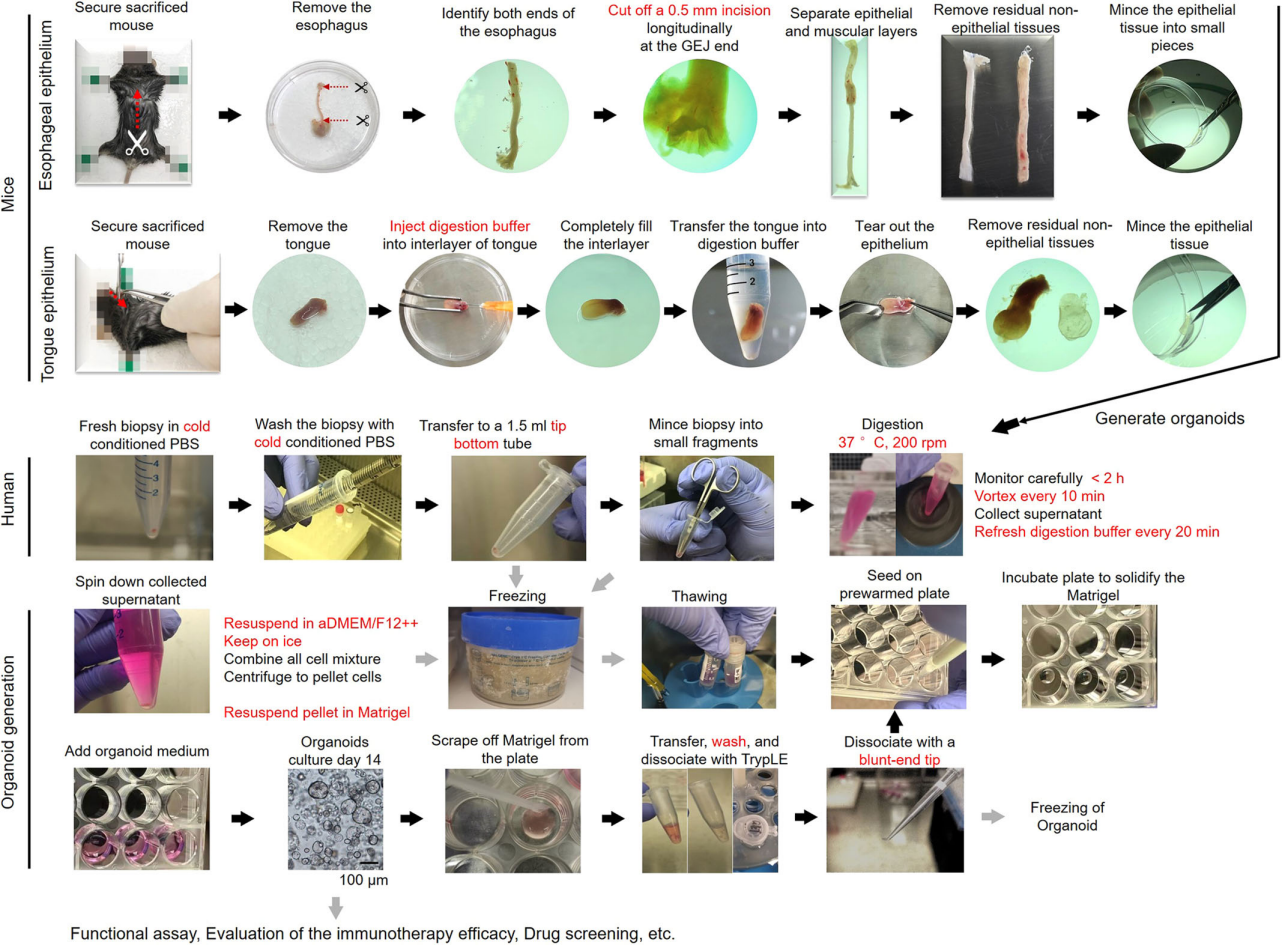
**Schematic overview of the procedure describing the establishment of organoids from primary tissue.** The upper panels show the isolation of normal esophageal and tongue epithelia from mouse. The lower panel describes sample collection from patient-derived esophageal squamous cell carcinoma (ESCC), gastroesophageal junction (GEJ) and Barrett's esophagus (BE) biopsy, and generation of organoids. Grey arrows represent optional steps. In the bottom row, a representative bright-field image of a GEJ organoid on culture day 14 is shown.

**Fig. 2.**
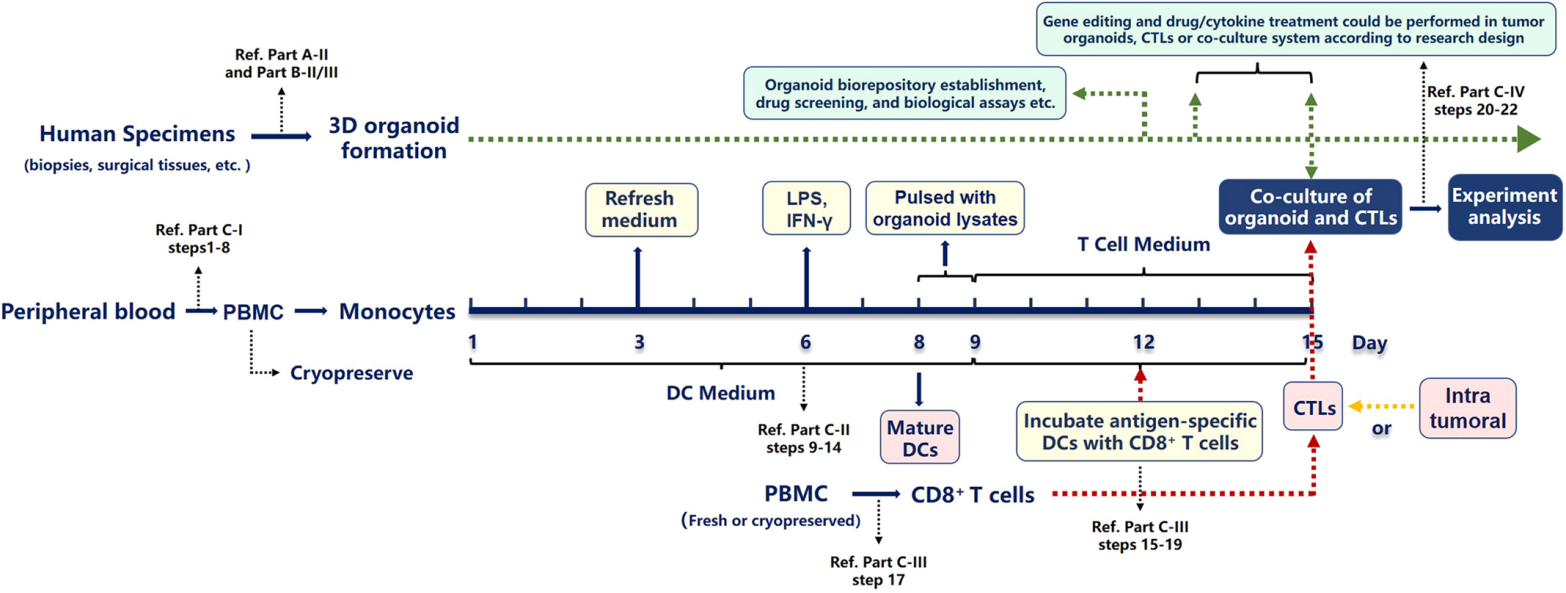
**Workflow of *ex vivo* co-culture system by incubating tumor organoids with tumor-antigen-specific cytotoxic T lymphocytes.** Primary tumor specimens are acquired from patients undergoing diagnostic endoscopy or surgical resection. Organoids are generated according to the procedure outlined in part A-II and part B-II/III. Cytotoxic T lymphocytes (CTLs) are collected from either autologous peripheral blood or tumors of patients and purified using the CD8^+^ T cell isolation kit. To induce a tumor-specific T cell response, mature dendritic cells (DCs) are obtained according to the procedure outlined in part C and pulsed with tumor organoid lysates. These DCs are then incubated with CD8^+^ T cells to present tumor-specific antigens. CD8^+^ T cells can specifically recognize tumor cells when co-cultured with patient-derived primary tumor organoids. This co-culture system could be used to assess the specific tumor killing effect, as well as the efficacy of immune checkpoint blockade treatments. To enhance tumoricidal effects, gene editing could be performed and drugs could be administrated in tumor organoids, CTLs or co-cultures according to users' research plan. PBMC, peripheral blood mononuclear cell.

Herein, we describe our optimized protocols to generate 3D organoid cultures from normal mouse esophageal and tongue epithelia, human gastroesophageal junction (GEJ) and abnormal human esophagus (ESCC and BE), as briefly alluded to in previous reports from our group and others (Driehuis et al., 2020; Kasagi et al., 2018; Kijima et al., 2019; Li et al., 2018; Liu et al., 2018; Trisno et al., 2018; Whelan et al., 2018; Zhang et al., 2018; Zhao et al., 2022). First, we provide a detailed illustration for the isolation of mouse esophageal and tongue epithelia (Fig. 1, Movies 1 and 2). Next, we explain how to generate 3D organoids from normal mouse epithelia, normal human GEJ, human ESCC and human BE (Fig. 1). Then, we describe generating 3D organoids from frozen human specimens as starting materials. In addition, we establish an *ex vivo* platform to study tumor-specific cytotoxic T lymphocyte (CTL)-mediated tumor killing and assess immunotherapeutic efficacy in co-cultured tumor organoids derived from patients with ESCC and from autologous cytotoxic CD8^+^ T cells (Fig. 2). Finally, we provide troubleshooting strategies relevant to this protocol.

### Applications of the protocol

This protocol can be applied to the investigation of EC and OC biology in general, as outlined below. (1) Generation of both normal esophageal and oral epithelial organoids as well as BE-derived organoids provides an excellent resource for studying *de novo* tumorigenesis, particularly the development of OC, ESCC and EAC. Cancer-driver genes can be introduced via genomic editing using CRISPR/Cas9 or lentivirus infection, which have been applied successfully to produce both murine ESCC- and human GEJ-derived transformed organoids (Wu et al., 2021; Zhao et al., 2022). In addition, this method can be extended to other squamous cell carcinomas from other organs, including the lung, head and neck, and cervix, which share similar pathological and molecular characteristics with oral and esophageal squamous epithelia. (2) Methods of establishing patient-derived organoids from ESCC, BE and GEJ can be applied to develop organoid biorepositories of ECs, as a strategy to preserve and extend studies of valuable clinical specimens. Moreover, patient-derived organoids are ideal for drug screening and therapeutic response prediction. (3) In immunology, the *ex vivo* co-culture system described herein enables researchers to study tumor-CD8^+^ T cell interactions and CD8^+^ T cell-mediated tumor killing at the level of individual patient samples. Furthermore, this system can be used to evaluate the sensitivity of cancers in individual patients to immunotherapies targeting cytotoxic CD8^+^ T cells, such as monoclonal antibodies against PD-1 (also known as PDCD1) and PD-L1 (or CD274). It can also allow researchers to identify effective drugs and rational combinations using chemotherapeutic agents, small-molecule inhibitors or ICB drugs through moderate- to high-throughput drug screening.

### Comparison with other approaches

Esophageal organoids were initially established from murine esophageal squamous stem cells (DeWard et al., 2014) using culture medium that is similar to that used for culturing intestinal organoids. Later studies suggested that several supplements are dispensable for the culture of normal murine esophageal organoids, such as N2, gastrin, FGF-10, N-acetylcysteine and HEPES (Zheng et al., 2021). Most studies focused on OC/EC initiation and regulation mechanisms have largely used murine models (Table 1). However, almost all of the current related publications describe only one tissue/species type. A comprehensive protocol detailing BE, GEJ and ESCC organoid culture, as well as their application in current immunotherapy, is still lacking. Here, (1) we provide an easy-to-follow, comprehensive protocol for growing organoids derived from normal epithelia of the murine tongue and esophagus, as well as samples of EC, BE and GEJ from human patients; and (2) our protocol includes novel and improved procedures associated with these cultures. For example, (i) we use optimized media to generate single-cell-derived organoids from normal murine tongue and esophagus, as well as human BE, GEJ and ESCC. Importantly, normal squamous epithelia and ESCC organoids share the same medium, as do BE and GEJ organoids, which can reduce workload. (ii) We also provide detailed production protocols of conditioned Wnt-3A, R-spondin-1 (RSPO1) media, and working concentrations for their commercial alternatives. Removal of the key growth factors Wnt-3A, R-spondin-1 and noggin does not affect the formation or growth of murine organoids derived from normal squamous epithelium (Karakasheva et al., 2020; Nakagawa et al., 2020). (3) We provide a freezing medium condition that can be used to cryopreserve fresh biopsies and tissue fragments from patients without significantly decreasing subsequent organoid-forming efficiency. This procedure can considerably facilitate the development of organoids from clinical samples that often require immediate lab processing.

**Table 1.**
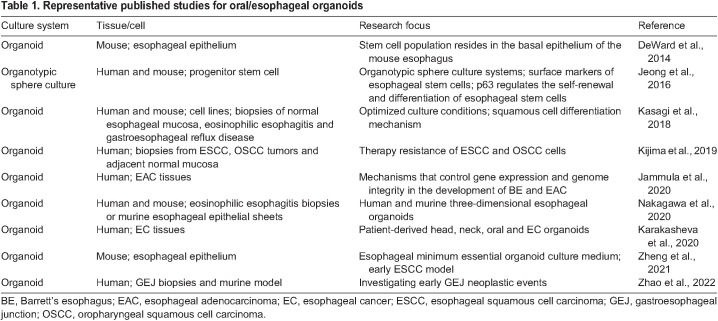
Representative published studies for oral/esophageal organoids

To assess the immunological response of human tumor tissues to *ex vivo* PD-1 blockade, several reports have used various systems, including air-liquid-interface organoids (Neal et al., 2018), microfluidic devices with organotypic tumor spheroids (Jenkins et al., 2018) and patient-derived tumor fragment platforms (Voabil et al., 2021). These studies have shown upregulated expression of cytotoxic proteins and secretion of cytokines, as well as intratumoral immune cell reinvigoration upon the treatment of ICB (Jenkins et al., 2018; Neal et al., 2018; Voabil et al., 2021). However, most of these experiments used mixed culture conditions containing various cell types, which may affect tumor-immune specificity (Deng et al., 2018; Jenkins et al., 2018). In another co-culture system, tumor organoids are incubated with peripheral blood mononuclear cells (PBMCs), which are stimulated with tumor organoids dissociated into single cells or with anti-CD3/anti-CD28 (Cattaneo et al., 2020; Dijkstra et al., 2018). In comparison, our approach uses standard antigen-presenting dendritic cells (DCs) and purified cytotoxic CD8^+^ T, which can respectively present tumor-specific antigens and execute direct antigen-dependent tumor killing. Additional gene manipulation or drug combination can be performed in the presence of tumor organoids and CD8^+^ T cells, facilitating the further investigation of ICB treatment.

## EXPERIMENTAL DESIGN

The protocols below are divided into three sections: (A) primary tissue collection and processing; (B) generation of organoids from both mouse and human primary tissues; and (C) co-cultures of tumor organoids and CTLs. Part A describes the isolation and processing of epithelial samples derived from the mouse esophagus and tongue, as well as from human ESCC, BE and GEJ. Part B details the initiation, passaging, cryopreservation and thawing of organoids derived from both mouse and human tissues. In part C, we describe the isolation of PBMCs, the generation of tumor specific-CTLs and the co-culture of tumor organoids and tumor-specific CD8^+^ T lymphocytes. Although part C describes the generation of esophageal organoids and its specific application to the evaluation of CD8^+^ T cell-targeted immunotherapy, this procedure can also be modified for other cancer types.

### Part A: epithelium collection and processing

For both murine and human organoid studies, collection of tissues must be performed in accordance with regulations and guidelines under approved protocols from a local regulatory body, e.g.*,* an institutional ethics committee or review board. For human organoid generation, collecting fresh, viable and pure (epithelium-rich) tissues is imperative to achieve high organoid-forming efficiency. For tumor samples, it is important to remove non-cancerous tissues and necrotic tissues as thoroughly as possible, as unpurified or necrotic tissues decrease organoid-forming capacity. In our hands, surgical specimens exhibited lower organoid-formation efficiency than fresh endoscopic biopsies. To maintain viability of tumor cells, fresh primary tissues should be collected and transferred into cold medium as soon as possible. Addition of the ROCK inhibitor Y-27632, fungizone, antibiotic-antimycotic or Primocin to all solutions enhances organoid growth and reduces the risk of fungal and/or bacterial contamination. Frozen clinical specimens typically have markedly reduced organoid formation rates; this challenge has presented a significant barrier to widespread human sample-based organoid culturing. Here, we describe our finding that primary tissue samples can be stored in a cell-freezing medium and placed in a liquid nitrogen tank for several months without significantly decreasing subsequent organoid-forming efficiency. Reagent preparation and expansion medium for the culture of different tissue-derived organoids are listed in the ‘Reagent setup’ section and Table 2, respectively.

**Table 2.**
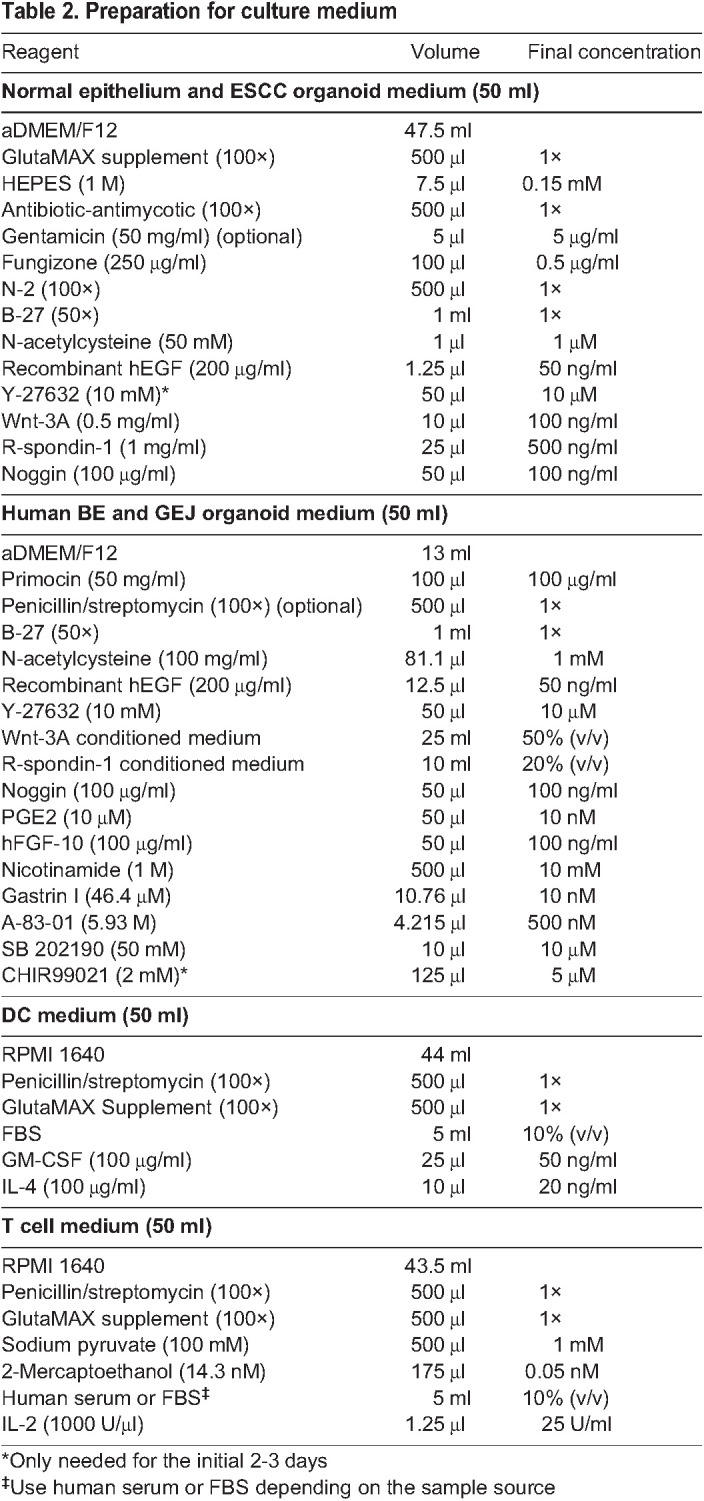
Preparation for culture medium

### Part B: generation of organoids from primary tissues

In this section of our protocol, we describe organoid initiation, passaging, cryopreservation and thawing. Due to different digestion conditions and culture media, we delineate organoid generation from normal murine epithelia, human ESCC, BE and GEJ, separately. In general, primary tissues are cleaned with Hank's balanced salt solution (HBSS) or PBS containing 10 μM Y27632, 2% (v/v) penicillin/streptomycin and 1× Primocin. Then, they are minced and digested with different digestion buffers, including collagenase type IV, collagenase type XI, trypsin or dispase. Harvested single cells are mixed with Matrigel and seeded in 24-well plates. The organoid medium is added in the 24-well plate once the Matrigel has completely solidified. For organoid passaging, the organoid-Matrigel droplets are removed with a mini-cell scraper or dissolved with cell recovery solution (digested by TrypLE Express Enzyme). Collected single cells can be seeded or used for other experiments. For all of the media used in this study, advanced Dulbecco's modified Eagle medium (aDMEM)/F12 works as a base medium. We use the same medium for the culture of human ESCC and murine epithelial organoids, except for the removal of Wnt-3A, R-spondin-1 and noggin for the culture of normal murine epithelial organoids. Herein, we provide both detailed production protocols of conditioned Wnt-3A, R-spondin-1 media, and working concentrations for their commercial alternatives to allow for more accessibility to these methods.

### Part C: co-culture of tumor organoids and CTLs

Immunotherapies using ICB combined with chemotherapies are the first-line treatment for advanced EC, as treatment with ICB drugs (such as pembrolizumab, nivolumab, toripalimab and camrelizumab) alone or in combination with chemotherapies improve overall survival and progression-free survival for advanced ESCC (Doki et al., 2022; Lu et al., 2022; Sun et al., 2021) and gastric, GEJ and esophageal adenocarcinoma (Janjigian et al., 2021). As CD8^+^ T cells are the key targets in ICB treatments, we describe a human *ex vivo* system by co-culturing EC organoids with CD8^+^ T cells (Fig. 2). CD8^+^ T cells can be collected from either autologous peripheral blood or tumors of patients with cancer and purified with commercial CD8^+^ T cell isolation kits. For antigen processing and presentation and induction of tumor-specific T cell responses, DCs are differentiated and matured using monocytes collected from autologous peripheral blood of patients by sequential incubation with GM-CSF (or CSF2), IL-4, lipopolysaccharide (LPS) and IFN-γ (IFNG), and pulsed with tumor cell lysates from isolated autologous tumor cells (Huang et al., 2018). These tumor-specific-antigen-pulsed DCs are then used to activate CD8^+^ T cells by co-incubation. Upon stimulation by DCs, CD8^+^ T cells can specifically recognize patient-derived primary tumor organoids when co-cultured with tumor cells. Users can dissociate parts of the organoids into single cells to calculate the number of tumor organoids and CD8^+^ T cells according to effector:target ratio. This *ex vivo* co-culture system can be used for antigen-dependent CD8^+^ T cell tumor-killing assays and for evaluation of the effect of ICB treatments based on users' research plans.

## MATERIALS AND EQUIPMENT

### Biological materials

#### Murine samples

C57BL/6JGpt mice (N000013) were purchased from Gem Pharmatech (Nanjing, China). All animal studies were performed according to the approval of the animal care regulations of Hefei Institutes of Physical Science, Chinese Academy of Sciences (Hefei, China).

#### Human ESCC samples

All ESCC samples were collected from patients who had provided informed consent, and were performed with the approval of the ethics committee of Hefei Cancer Hospital, Chinese Academy of Sciences (Hefei, China).

#### Human BE and GEJ biopsies

In accordance with Johns Hopkins University Institutional Review Board standards and guidelines, primary human GEJ and BE biopsy samples were acquired from patients undergoing diagnostic endoscopy at the Johns Hopkins Hospital with written informed consent.

#### Cell lines

L Wnt-3A cell line (CRL-2647) producing Wnt-3A conditioned medium was purchased from American Type Culture Collection. Cultrex HA-R-spondin-1-Fc 293T cell line (3710-001-01) producing R-spondin-1 conditioned medium was purchased from Bio-Techne.

### Reagents

- PBS (Corning, 21-040-CV)
- Hank's balanced salt solution (HBSS) (Thermo Fisher Scientific, 14175079)
- Trypan Blue cell stain (Thermo Fisher Scientific, 15250061)
- Soybean trypsin inhibitor (STI) (Millipore, T9128-1G)
- Penicillin/streptomycin mixture (Quality Biological, 120-095-721)
- Antibiotic-antimycotic (Thermo Fisher Scientific, 15240062)
- DMEM (Corning, 10-013-CV)
- Fetal bovine serum (FBS) (Corning, 35-0.1-CV)
- G418, 50 mg/ml (Invitrogen, 10131035)
- Zeocin, 100 mg/ml (Invitrogen, 45-0430)
- Recovery Cell Culture Freezing Medium (Thermo Fisher Scientific, 12648-010-100ML)
- DNase I (Sigma-Aldrich, 10104159001)
- Collagenase, type IV (Thermo Fisher Scientific, 14175079-1G)
- Collagenase, type XI from *Clostridium histolyticum* (Sigma-Aldrich, C7657-100MG)
- Dispase (Corning, 354235-100ML)
- Dispase II (Thermo Fisher Scientific, 17105041-5G)
- CHIR99021 (Sigma-Aldrich, SML1046-5MG)
- PGE2 (Sigma-Aldrich, P0409-1MG)
- Recombinant human FGF-10 (hFGF-10) (Peprotech, 100-26, 250UG)
- hEGF (Sigma-Aldrich, E9644-0.1MG; Peprotech, AF-100-15)
- Recombinant murine Wnt-3A V2 (Novoprotein, C18K, 50UG)
- Recombinant murine noggin (Peprotech, 250-38, 100UG)
- Recombinant human R-spondin-1 (Peprotech, 120-38, 20UG)
- N-acetyl-L-cysteine (Sigma-Aldrich, A9165-5G)
- Nicotinamide (Sigma-Aldrich, N0636-100G)
- [Gly^18^]-gastrin 1-17 human (Sigma-Aldrich, SCP0150-500UG)
- A-83-01 (Sigma-Aldrich, SML0788-5MG)
- SB 202190 (Sigma-Aldrich, S7067-25MG)
- Y-27632 2HCl (Selleck Chemicals, S1049-10MG)
- Primocin (InvivoGen, ant-pm-1-500MG)
- B-27 supplement (50×), serum free (Thermo Fisher Scientific, 17504044)
- TrypLE Express Enzyme (1×), no phenol red (Thermo Fisher Scientific, 12604013)
- Trypsin-EDTA (Thermo Fisher Scientific, 15400054)
- Bovine serum albumin (BSA) solution (Sigma-Aldrich, A8412-100ML)
- Advanced DMEM (aDMEM)/F-12 (Thermo Fisher Scientific, 12634010)
- GlutaMAX supplement, 100× (Thermo Fisher Scientific, 35050061)
- Gentamicin (Solarbio, G1870)
- N-2 supplement, 100× (Thermo Fisher Scientific, 17502048)
- Fungizone (Thermo Fisher Scientific, 15290018)
- HEPES, 1 M (Thermo Fisher Scientific, 15630080)
- Matrigel matrix (Corning, 356231)
- DC isolation kit (Miltenyi, 130-094-487)
- MojoSort™ Human CD8 T Cell Isolation Kit (Biolegend, 480129)
- MojoSort™ Human CD8 Nanobeads (Biolegend, 480108)
- Human serum (from human male AB plasma; Sigma-Aldrich, H3667)
- 2-Mercaptoethanol (Thermo Fisher Scientific, 21985023)
- Sodium pyruvate (Thermo Fisher Scientific, 11360070)
- IFN-γ (PeproTech, 300-02)
- IL-2 (PeproTech, 200-02)
- IL-4 (PeproTech, 200-04)
- GM-CSF (PeproTech, 300-03)
- Ficoll-Paque (Cytiva, 17544202)
- DMSO (Sigma-Aldrich, D2650-100ML)
- CellTrace™ Violet Cell Proliferation Kit (Thermo Fisher Scientific, C34557)
- Propidium iodide (Solarbio, C0080)
- Lipopolysaccharide (LPS) (Sigma-Aldrich, L2880-10MG)
- Dulbecco's phosphate-buffered saline (Solarbio, P1022)
- Anti-TP63 (1:200, Cell Signaling Technology, 13109S)
- Anti-SOX2 (1:1000, Active Motif, 39823)
- Anti-Ki67 (1:200, Abcam, ab16667)
- Anti-keratin 14 (1:200, Proteintech, 10143-1-AP)
- Anti-keratin 13 (1:100, Proteintech, 10164-2-AP)
- Anti-involucrin (1:600, Proteintech, 28462-1-AP)
- Anti-CD8-FITC (1:50, Biolegend, 301060)

### Equipment

- Precision Scientific GCA water bath (American Laboratory Trading, 66557/23)
- Forma 430 orbital shaker (Thermo Forma, 20470)
- Desktop constant temperature oscillator (PeiYing, THZ-D)
- Cell analyzer (Countstar, IC1000)
- Chamber slide (Countstar, 12-0005-50)
- SterilGARD Class II Type A2 biosafety cabinet (The Baker Company, Inc., SG304)
- Heracell 150i CO_2_ incubator (Thermo Fisher Scientific, 50116047)
- Cell counter (Countstar, IC1000)
- Nikon Eclipse TS100 inverted routine microscope (Marshall Scientific, 301196)
- MyFuge Mini Centrifuge (Benchmark Scientific, C1008-C)
- Refrigerated centrifuge (Thermo Fisher Scientific, ST16R)
- Cryogenic freezing container (Thermo Scientific Nalgene, 5100-0001)
- Angulus oris and dissecting scissors (SanYou)
- Vertical mixer (Yooning, VM-100)
- Electric suction apparatus (Yuwell, 7A-23D)
- Liquid nitrogen tank (Thermolyne Locator 4 Cryostorage, TH-L4)
- Eppendorf safe-lock microcentrifuge tubes, 1.5 ml (Sigma-Aldrich, T9661)
- Portable Pipet-Aid (Drummond Scientific, 4-000-100)
- Falcon conical centrifuge tubes, 15 ml (Corning, 352096)
- Falcon conical centrifuge tubes, 50 ml (Corning, 352070)
- TipOne filter tips, 10 µl (USA Scientific, 1120-3810)
- TipOne filter tips, 20 µl (USA Scientific, 1123-1810)
- TipOne filter tips, 200 µl (USA Scientific, 1120-8810)
- TipOne filter tips, 1000 µl (USA Scientific, 1126-7810)
- Falcon disposable polystyrene serological pipette, 5 ml (Corning, 352098)
- Falcon disposable polystyrene serological pipette, 10 ml (Corning, 357551)
- Falcon disposable polystyrene serological pipette, 25 ml (Corning, 357525)
- Sterile vacuum filter units, 50 ml (Millipore Sigma, SCGP00525)
- Mini cell scrapers (UnitedBioSystems, MCS-200)
- Falcon Clear Flat Bottom TC-treated Multiwell Cell Culture Plate, 24-well (Corning, 353047)
- Falcon Cell Clear Flat Bottom TC-treated Multiwell Culture Plate, 6-well (Corning, 353934)
- Eppendorf cell culture dishes, 60×15 mm (Sigma-Aldrich, EP0030701011)
- Cell culture flask, t-75, surface: standard, filter cap (SARSTEDT, 83.3911.002)
- 1 ml syringe (Kangyeda)
- 5 ml syringe (ZYMM)
- Steriflip filter unit (0.22 µm pore size) (Millipore, SLGP033RB)
- 96-well U-bottom plate (NEST Scientific, 701111)

### Reagent preparation

#### Conditioned PBS

Supplement PBS with 10 μM ROCK inhibitor Y27632, 2% (v/v) penicillin/streptomycin and 1× Primocin.

#### Collagenase, type XI from *Clostridium histolyticum*

Mix 100 mg of collagenase XI in 10 ml of 50 mM TES buffer, pH 7.4, containing 0.36 mM calcium chloride to make a 10 mg/ml stock solution. Make 500 μl aliquots and store at −20°C.

#### Dispase II

Dissolve 60 mg of dispase II in 6 ml of PBS to make a 10 mg/ml stock solution. Make 600 μl aliquots and store in a dark place at 4°C for ≤24 months.

#### Digestion buffer I

Supplement HBSS with 1% (v/v) penicillin/streptomycin, 50 U/ml dispase, 20 mg/ml collagenase IV and 10 μM Y-27632.

#### Digestion buffer II

Supplement DMEM with 2.5% (v/v) FBS, 1% (v/v) penicillin/streptomycin, 1 mg/ml collagenase type XI and 120 μg/ml dispase type II stock solution. Store at −20°C for ≤3 months.

#### aDMEM/F12++

Supplement aDMEM/F12 with 10 μM ROCK inhibitor Y27632 and 1% (v/v) penicillin/streptomycin.

#### aDMEM/F12+++

Supplement aDMEM/F12 with 10 μM ROCK inhibitor Y27632, 5 μM CHIR99021 and 1% (v/v) penicillin/streptomycin.

#### Wnt-3A conditioned medium

Divide one vial of L Wnt-3A cells into a T-75 flask with 12 ml of DMEM containing 10% (v/v) FBS and 0.4 mg/ml G418. Incubate the culture at 37°C, 5% (v/v) CO_2_. When the cell confluency reaches 90%, split the cells in a 1:10 ratio in 12 ml of culture medium (without G418) in T-75 flasks and allow the cells to grow for 5 days. Collect the medium and filter it through a 0.22 μm filter. Store this first batch of medium at 4°C. Add 12 ml of fresh DMEM containing 10% (v/v) FBS and culture for another 3 days. Collect the medium and sterilize by filtering. Combine the first and second batches of media. Make 50 ml aliquots and store at −20°C for ≤12 months.

#### R-spondin-1 conditioned medium

Divide one vial of HA-R-spondin-1-Fc 293T cells into a T-75 flask with 12 ml of DMEM containing 10% (v/v) FBS and 300 μg/ml zeocin. Incubate the culture at 37°C, 5% (v/v) CO_2_. When the cell confluency reaches 90%, split the cells in a 1:10 ratio in 12 ml of culture medium (without zeocin) in T-75 flasks and allow the cells to grow for 5 days. Collect the medium and filter through a 0.22 μm filter. Store this first batch of medium at 4°C. Add 12 ml of fresh DMEM containing 10% (v/v) FBS and culture for another 3 days. Collect the medium and sterile by filtering. Combine the first batch and second batch of media. Make 10 ml aliquots and store at −20°C for ≤12 months.

#### CHIR99021

Dissolve 5 mg of CHIR99021 in 5.372 ml of DMSO to make a 2 mM stock. Make 250 μl aliquots and store at −20°C.

#### PGE2

Dissolve 1 mg of PGE2 in 1 ml of absolute ethanol and store this stock in a dark place at 4°C for ≤36 months. Further dissolve 2.5 μl of 1 mg/ml PGE2 in 700 μl of 1% (w/v) BSA to make a 10 mM stock solution. Make 100 μl aliquots and store at −20°C for ≤1 month.

#### hFGF-10

Dissolve 250 μg of hFGF-10 in 2.5 ml of 5 mM sodium phosphate, pH 7.4, to make a 100 μg/ml stock. Make 100 μl aliquots and store at −20°C.

#### hEGF

Dissolve 100 μg of hEGF in 500 μl of PBS to make a 200 μg/ml stock. Make 25 μl aliquots and store at −20°C.

#### Wnt-3A

Dissolve 50 μg recombinant human Wnt3A V2 in 100 μl distilled water to form 0.5 mg/ml storage solution, and dilute the storage solution 1:5000 for use (100 ng/ml). Make 5 μl aliquots and store at −80°C.

#### R-spondin-1

Dissolve 20 μg recombinant human R-spondin-1 in 20 μl distilled water to form 1 mg/ml storage solution, and dilute the storage solution 1:2000 for use (500 ng/ml). Make 5 μl aliquots and store at −80°C.

#### Noggin

Dissolve 100 μg of noggin in 1 ml of 10 mM PBS to make a 100 μg/ml stock. Make 100 μl aliquots and store at −20°C for ≤12 months.

#### N-acetylcysteine

Dissolve 200 mg of N-acetylcysteine in 2 ml of H_2_O to make a 100 mg/ml stock. Make 162.2 μl aliquots and store at −20°C.

#### Nicotinamide

Dissolve 1221.2 mg of nicotinamide in 10 ml of H_2_O to make a 1 M stock. Make 1 ml aliquots and store at −20°C.

#### Gastrin I

Dissolve 1 mg of Gastrin I in 10 ml of H_2_O to make a 46.4 μM stock. For a final concentration of 10 nM, use 21.5 μl of this stock to make 100 ml of conditioned organoid medium.

#### A-83-01

Dissolve 5 mg of A-83-01 in 2 ml of DMSO to make a 5.93 M stock. For a final concentration of 500 nM, use 8.43 μl of this stock to make 100 ml of conditioned organoid medium.

#### SB 202190

Dissolve 25 mg of SB 202190 in 1.509 ml of DMSO to make a 50 mM stock. Make 20 μl aliquots and store at −20°C.

#### Y-27632

Dissolve 10 mg of Y27632 in 3. 3.1225 ml of H_2_O to make a 1 mM stock. Make 50 μl aliquots and store at −20°C.

#### Collagenase IV

Dissolve 250 mg of collagenase IV into 5 ml of HBSS to make a 50 mg/ml stock. Make 1 ml aliquots and store at −20°C. Thaw in a water bath at 37°C for 1 min prior to use.

#### Dispase

Store undiluted dispase aliquots (1 ml) up to 3 months at −20°C. Thaw in a water bath at 37°C for 1 min prior to use.

#### Fungizone

Store undiluted fungizone aliquots (1 ml) up to 12 months at −20°C. Thaw in a water bath at 37°C for 1 min prior to use.

#### STI

Dissolve 250 mg of STI in 1000 ml Dulbecco's phosphate-buffered saline (250 mg/l) and filter-sterilize via a 1000 ml filter cup. Dispense aliquots into 50 ml polypropylene tubes, which can be stored up to 6 months at 4°C.

#### IFN-γ

Initially reconstitute in water to 1.0 mg/ml. Store at 2 to 8°C for up to 1 week, or aliquot (20 µl) and store up to 3 months at −80°C.

#### GM-CSF

Initially reconstitute in water to 100 μg/ml. Store at 2 to 8°C for up to 1 week, or aliquot (20 µl) and store up to 3 months at −80°C.

#### IL-2

Initially reconstitute in 100 mM acetic acid to 1.0 mg/ml (corresponding to a specific activity of ≥1×10^7^ U/mg). Store at 2 to 8°C for up to 1 week, or aliquot (20 µl) and store up to 3 months at −80°C.

#### IL-4

Initially reconstitute in water to 100 μg/ml. Store at 2 to 8°C for up to 1 week, or aliquot (20 µl) and store up to 3 months at −80°C.

#### Culture medium

To prepare culture medium, mix all the growth factors and conditioned medium as listed in Table 2. Filter the complete medium though the Steriflip filter unit (0.22 µm pore size) and collect the filtrate in the attached 50 ml centrifuge tube. The medium can be stored at 4°C for ≤2 weeks.

## PROCEDURE

See Table 3 for an overview of the protocol timeline.

**Table 3.**
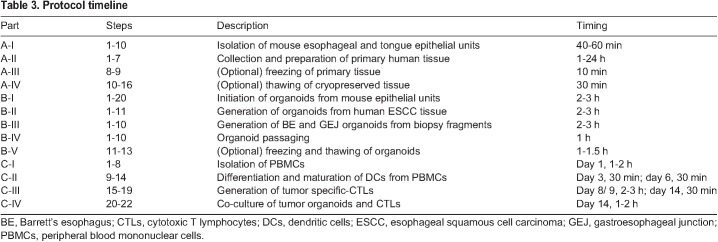
Protocol timeline

### Part A: epithelium collection and processing

#### I. Isolation of mouse esophageal and tongue epithelial units

**Timing: 40-60 min**

1. Euthanize mice by isoflurane (in a desiccator jar used within the approved fume hood) followed by cervical dislocation.
2. Wet the fur of the sacrificed mouse using 75% ethanol and secure its limbs by pushing pins with the abdomen upward.

##### For esophagus

3. Cut away the fur along the anterior median line of the mouse thorax to open the thoracic cavity using dissecting scissors.
4. Remove the esophagus using the forceps and put it flat on the inside of the lid of a 60 mm Petri dish with a few drops of HBSS containing penicillin/streptomycin.
5. Identify both ends of the esophagus with a stereoscope (upper end, esophageal-pharyngeal junction; lower end, GEJ) and cut off a 0.5 mm incision longitudinally at the GEJ end using dissecting scissors.
  **CRITICAL:** Two obvious layers can be observed at the incision site of the GEJ end. The inner layer is a multi-layered epithelium and the outer one is a muscular layer.
6. Peel the epithelial and the muscular layers apart longitudinally from the incision with two sharp-end forceps to obtain the epithelial units.
  **CRITICAL:** It is easier to peel from the lower side of the esophagus (GEJ end), as the lumen at the GEJ is wider relative to that at the esophageal-pharyngeal junction. Remove residual non-epithelial tissues at both the upper and lower ends of the esophagus using forceps and scalpel.

##### For tongue

7. Cut along the angulus oris to the pharynx of the sacrificed mouse to open the oral cavity with dissecting scissors. Remove the tongue from the root of the tongue by clipping the tip of the tongue and putting it flat on the inside of the lid of a 60 mm Petri dish (the edge of the Petri dish is too high to operate). Add a few drops of HBSS containing penicillin/streptomycin to prevent tissue drying and to avoid bacteria contamination.
8. Clamp the excised tongue with blunt-end forceps and inject 500 µl of digestion buffer I between the epithelial and the muscle layers of the tongue with a 1 ml syringe needle.
  **CRITICAL:** Do not pierce the epithelial layer when adding digestion buffer I with the needle. Keep the epithelium intact. Slowly pull the needle back while injecting to ensure that the digestion buffer I fills in the whole interlayer.
9. Transfer the stuffed tongue into a 15 ml conical tube containing 3 ml of digestion buffer I and place the tube among the springs of a shaker at a slight angle (∼15°). Digest the tissue at 37°C for 20-30 min. Once the epithelial and muscle layers have completely separated, proceed to the next step.
  **! CAUTION:** Digestion time is about 20-30 min. Over-digestion may significantly impair cell viability.
  **CRITICAL:** Keeping the tube at a slight angle for tissue digestion at a 37°C shaker facilitates separation of the epithelium from the muscle layers of the tongue.
10. Prepare both the blunt-end and sharp-end forceps. Using the blunt-end forceps, hold the epithelium at the tip of the tongue and use the sharp-end forceps to clamp the muscle layer from the back of the tongue. Gently tear toward both ends to separate the two layers.
  **CRITICAL:** Try to tear both epithelial and muscle layers simultaneously, as multiple tearing can result in the epithelium breaking and, consequently, the undesired inclusion of non-epithelial cells.

#### II. Collection and preparation of primary human tissues (BE, GEJ and ESCC)

**Timing: 1-24** **h**

Collecting fresh, viable and pure (epithelium-rich) tissues is imperative to achieve high organoid-forming efficiency.

1. Collect fresh patient endoscopic tissues in 10 ml of ice-cold PBS or HBSS containing 10 μM ROCK inhibitor Y27632, 2% (v/v) penicillin/streptomycin and 1× Primocin to ensure cell viability and inhibit microbe growth. Keep the tissues at 4°C, and further processing should ideally be performed as soon as possible.
  **PAUSE POINT:** Tissue pieces may be stored in the conditioned PBS within 24 h without significantly decreasing organoid-forming efficiency.
  **Troubleshooting:** see Table 4.
2. Transfer the tissue pieces into a new 15 ml conical tube containing 10 ml of ice-cold conditioned PBS. Wash the samples by pipetting up and down with a 10 ml serological pipette at least ten times. Keep the tube still to allow the tissue pieces to settle at the bottom.
  **CRITICAL:** Small tissue pieces and organoids (especially when mixed with Matrigel) are likely to adhere to the walls of tips and tubes. To prevent this as much as possible, for all subsequent steps, rinse all serological pipettes and filter tips that directly contact tissues or organoids with DMEM containing 10% (v/v) FBS medium before use. Use low-binding-affinity tubes to avoid cell loss.
  **Troubleshooting:** see Table 4.
3. Aspirate the supernatant with a 10 ml pipette and add 10 ml of ice-cold conditioned PBS.
4. Repeat the washing steps 2 and 3 at least five times until the supernatant is debris-free to avoid bacterial contamination. Alternatively, wash the tissue three times on a rotator, 5 min for each wash.
  **Troubleshooting:** see Table 4.
5. Endoscopy biopsies usually have less fat and muscle tissue compared with the surgically resected specimens. If necessary, strip the underlying muscle layer off by using fine scissors under a stereomicroscope after washing.
  **Note**: For surgically resected specimens, place the tissue in a 60 mm Petri dish and add ice-cold PBS containing 2% (v/v) penicillin/streptomycin (just immersing the tissue). Remove adipose, muscle and necrotic tissues, as well as blood vessels with fine dissecting scissors and forceps. Transfer the tissue into a 1.5 ml centrifuge tube and wash the tissue three times with PBS on a rotator.
  **CRITICAL:** To remove non-target tissues, wash the tissue by pipetting up and down with a 1 ml tip five to ten times when changing PBS.
6. Transfer the tissue pieces to a 1.5 ml Eppendorf tube and add a small volume (just immersing the tissue pieces) of aDMEM/F12++ medium. Keep the tubes on ice and thoroughly mince the samples into small fragments <1 mm^3^ in size using fine, straight-point micro-dissecting scissors. Sterilize the scissors before use and change them for different samples. These measures reduce tissue loss and improve crypt isolation efficiency.
7. Shortly spin down the tissue fragments and discard the supernatant completely. Keep part of the tissue fragments for further histological, molecular and biochemical analyses.

**Table 4.**
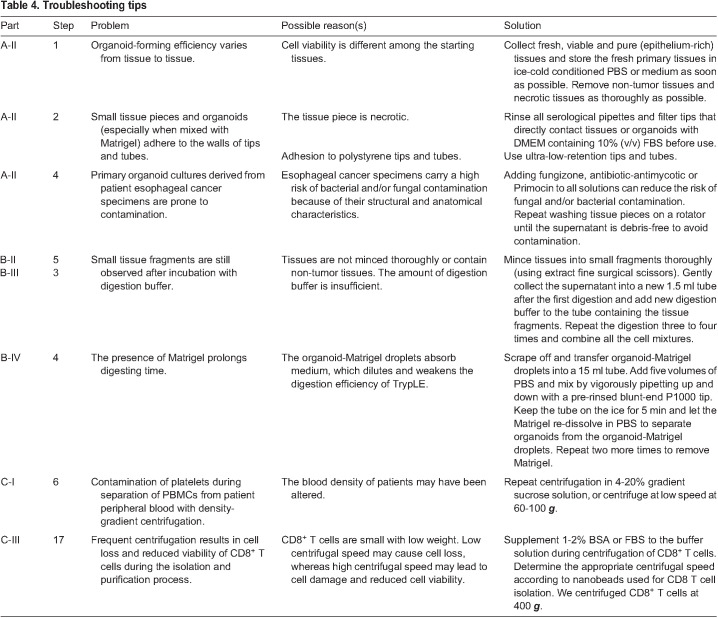
Troubleshooting tips

#### III. (Optional) freezing of primary tissue

**Timing: 10 min**

8. Transfer the tissue or tissue pieces into a cryogenic vial containing 1 ml of cell freezing medium.
9. Place cryogenic vials in a cryogenic freezing container and store at −80°C for 24 h, then transfer the vials to a liquid nitrogen tank for long-term storage.
  **PAUSE POINT:** Cryopreserved tissue may be stored in a liquid nitrogen tank for several years without significantly decreasing organoid-forming efficiency.

#### IV. (Optional) thawing of cryopreserved tissue

**Timing: 30 min**

10. Prepare 10 ml of aDMEM/F12+++ medium for each cryopreserved tissue vial in a water bath at 37°C.
  **CRITICAL:** The addition of Y27632 and CHIR99021 ameliorate cell recovery and improve organoid-forming efficiency.
11. Collect the cryopreserved tissue vials from the liquid nitrogen tank quickly and keep them on dry ice in transit to the water bath.
12. Rapidly thaw the vial in a 37°C water bath and carefully monitor the thawing. Do not allow the freezing medium to completely thaw. Remove the vial from the water bath when a small chunk of ice remains inside.
13. Quickly transfer the contents of the vial to a 15 ml tube. Then, add 9 ml of pre-warmed aDMEM/F12+++ medium dropwise to the tube to avoid osmotic shock.
14. Centrifuge the tube at 400 ***g*** for 3 min. Discard the supernatant containing DMSO completely.
15. Resuspend the tissue pieces in 1 ml of pre-warmed medium and transfer them to a 1.5 ml tube.
16. Centrifuge the tube at 400 ***g*** for 3 min and discard the supernatant completely.

### Part B: generation of organoids from primary tissues

#### I. Initiation of organoids from mouse epithelial units

**Timing: 2-3 h**

1. Place the collected epithelial tissue into a 1.5 ml centrifuge tube with sterile forceps.
2. Wash the tissue three times in HBSS containing penicillin/streptomycin. Each wash should be at least 5 min.
3. Transfer the epithelial tissue into a 60 mm Petri dish and keep the Petri dish on ice. Mince the tissue into small fragments <1 mm^3^ in size using sterile dissecting scissors.
4. Quickly transfer the minced tissue fragments into a 1.5 ml centrifuge tube with 1 ml collagenase IV (5 mg/ml), and then gently shake and incubate at 37°C for 15 min.
  **CRITICAL:** Thermomixer C or a shaker incubator is highly recommended as shaking at 37°C will enhance the digestion process. The incubation time could be extended by supplementation with the ROCK inhibitor Y-27632 (10 µM) in accordance with the size of the tissue fragments but should not exceed 1 h.
5. Centrifuge at 600 ***g*** for 3 min and discard the supernatant.
6. Resuspend the tissue fragments in 1 ml of HBSS containing penicillin/streptomycin.
7. Spin down the cells at 600 ***g*** for 3 min and discard the supernatant.
8. Resuspend the tissue fragments with 1 ml of pre-warmed 0.25% trypsin-EDTA supplemented with DNase I (10 U/ml) and incubate at 37°C for 10 min with shaking at 200 rpm.
9. Centrifuge at 600 ***g*** for 3 min and discard the supernatant.
10. Resuspend the trypsinized cells with 1 ml of STI.
11. Filter the cell suspension over a 70 μm cell strainer into a 50 ml conical tube. Rinse the strainer with 4 ml of STI.
  **CRITICAL:** Residual tissue fragments could be mashed using the rubber plunger of a 1 ml syringe and rinsed with STI through the cell strainer to increase single-cell yields.
12. Centrifuge at 600 ***g*** for 3 min and discard the supernatant.
13. Resuspend the cells in 100 μl HBSS containing penicillin/streptomycin and transfer into a 1.5 ml centrifuge tube. Keep on ice.
14. Count the cells with the cell counter by diluting 10 μl cell suspension in Trypan Blue.
15. Pre-warm the 24-well plate at 37°C for >1 h.
16. Adjust the cell concentration and resuspend the pellet with 1:4 PBS/Matrigel mixture.
17. Plate 10,000-20,000 cells into the center of each well of a 24-well plate in a 50 μl PBS/Matrigel droplet.
18. Place the 24-well plate in a 37°C incubator.
  **Optional:** After 5 min, invert the plate and incubate for another 25 min to allow the cell/Matrigel droplets to solidify completely.
  **CRITICAL:** Thaw and keep the Matrigel on ice always. Resuspend the cell pellet with PBS before adding Matrigel to help with mixing, and mix the cells with PBS/Matrigel carefully and gently to avoid any bubbles and solidification. Inverting the plate shortly during the solidification of cell/Matrigel droplets sometimes prevents the contact of organoids with the plate.
19. Slowly add 500 μl of murine epithelial organoid medium to each well and supplement with 10 μM ROCK inhibitor Y-27632 and 1% (v/v) penicillin/streptomycin to increase single cell viability and prevent bacterial contamination.
  **CRITICAL:** Add PBS into the unused wells surrounding cell-seeded wells to prevent evaporation of the medium.
20. Change the medium every 2 days.
  **CRITICAL:** Generally, about 10 days after seeding, epithelial organoids derived from the tongue or esophagus can be observed and used for downstream processes, including passaging, cryopreservation, and cellular and molecular biology experiments [e.g. fluorescence *in situ* hybridization, immunohistochemistry (IHC), PCR, etc.].

**Note:** All the reagents and tools including medium, supplements, forceps and scissors should be sterilized prior to use. Use cell culture-grade or disposable plasticware and glassware.

#### II. Generation of organoids from human ESCC tissues

**Timing: 2-3 h**

1. Transfer the tissue to the inner edge of a 60 mm Petri dish once the tissue becomes white and blood-free.
2. Add 100 μl collagenase IV (5 mg/ml) and thoroughly mince the tissue into small fragments <1 mm^3^ in size (when the solution becomes cloudy and blurry) using fine dissecting scissors.
3. Resuspend the minced tissue fragments with 400 μl collagenase IV, and transfer into a 1.5 ml centrifuge tube.
4. Repeat step 3 once to collect all the minced tissue fragments.
  **CRITICAL:** The amount of collagenase IV (1 ml, 5 mg/ml) used applies to two biopsy tissues. The amount of collagenase IV should be appropriately adjusted according to the size of the collected tissue.
5. Place the tube horizontally in an incubator shaker and incubate the tissue fragments for 1 h at 37°C with shaking at 200 rpm. Vortex the tube for 3-5 s every 15 min.
  **CRITICAL:** To fully digest the tissue, two choices can be considered. (i) Extension of incubation time: the incubation time of collagenase IV can be extended by supplementing with 10 μM ROCK inhibitor Y-27632, but the total digestion time should not exceed 2 h. (ii) Step-by-step digestion: gently collect the supernatant to a 15 ml conical tube after incubation and digest the remaining tissue fragments by adding new collagenase IV. Repeat these digestion-collection processes two to three times and combine all digested tissue fragments into the 15 ml conical tube.
  **Troubleshooting:** see Table 4.
6. Collect all digested tissue fragments, centrifuge at 600 ***g*** for 3 min, and discard the supernatant.
7. Resuspend the pellet with HBSS containing penicillin/streptomycin.
8. Centrifuge at 600 ***g*** for 3 min and discard the supernatant.
9. Add 1 ml of pre-warmed 0.25% trypsin supplemented with DNase I (10 U/ml).
10. Incubate in an incubator shaker at 37°C for 10 min with horizontal shaking at 200 rpm.
11. For subsequent steps, refer to steps 9-20 of part B-I: ‘Initiation of organoids from mouse epithelial units’.

#### III. Generation of BE and GEJ organoids from biopsy fragments

**Timing: 2-3 h**

1. Add 1 ml of pre-warmed fresh digestion buffer II to the 1.5 ml tube containing the tissue fragments (from step 7 of part A-II or step 16 of part A-IV). Mix thoroughly by vortexing for 5 s.
2. Incubate the tubes at 37°C with shaking at 200 rpm. Vortex the tubes every 10 min.
3. After incubation for 20 min, stand the tube still until the tissue fragments settle at the bottom. Gently collect the supernatant to a new 1.5 ml tube and add new digestion buffer II to the tube containing the tissue fragments.
  **Troubleshooting:** see Table 4.
4. Centrifuge the tube containing the supernatant at 400 ***g*** for 2 min, aspirate the supernatant without disturbing the pellet and add 1 ml of aDMEM/F12++. Keep the tube on ice.
5. Repeat steps 1-4 at least three times.
  **CRITICAL:** For BE and GEJ organoids, the digestion time is about 1-2 h. As over-digestion may significantly decrease outgrowth efficiency, regularly monitor the digestion by checking the 1.5 ml tube under a microscope. A mixture of cell clusters (three to 15 cells) should be observed.
6. Combine the cell mixture from the same sample into a 15 ml tube. Spin the cells at 400 ***g*** for 3 min at 4°C and discard the supernatant completely.
7. Resuspend the pellet with Matrigel. Use a ratio of cells to Matrigel that will allow 10,000 cells in 100 µl of Matrigel.
  **CRITICAL:** Carefully and gently mix the pellet with Matrigel on ice to avoid any bubbles and solidification.
8. Dispense 50-100 µl of the cell-Matrigel suspension into the center of each well of a 24-well plate using a P200 tip.
  **CRITICAL:** Pre-warm the plate at 37°C for over 1 h. Pre-warming helps with the polymerization of cell-Matrigel droplets.
9. Place the plate in a 37°C incubator for 10 min to solidify the cell-Matrigel droplets.
10. Slowly add 500 µl of pre-warmed BE or GEJ organoid medium (growth factors are listed in Table 2) supplemented with 5 µM CHIR99021 to each well. Incubate the plate at 37°C and 5% (v/v) CO_2_. Observe the organoids daily and refresh the medium at minimum every 2-3 days.
  **CRITICAL:** For the first few passages, it is essential to add CHIR99021 and penicillin/streptomycin in the organoid culture medium.

#### IV. Organoid passaging

**Timing: 1 h**

1. Remove the medium carefully without disturbing the organoid-Matrigel droplets. Scrape off the droplets with a mini cell scraper and transfer them into a 1.5 ml tube (less than 200 µl in each tube) or into a 15 ml tube (less than 2 ml in each tube).
2. Add five volumes (treat the volume of the organoid-Matrigel mixture as one volume) of PBS into the tubes and mix well by pipetting up and down. Centrifuge at 500 ***g*** for 3 min at room temperature and discard the supernatant completely.
3. Repeat step 2 two more times.
  **CRITICAL:** The organoid-Matrigel droplets absorb the medium, which will dilute and weaken the digestion efficiency of TrypLE. Washing with PBS helps eliminate the extra medium and avoids a long digestion time, especially when digesting a large volume of organoid-Matrigel mixture. TrypLE could be replaced by 0.25% trypsin supplemented with DNase I to obtain single cells according to different research requirements.
4. Add 2.5 volumes of TrypLE including 10 μM Y27632 to the organoid-Matrigel mixture. Mix by vigorously pipetting up and down (eight to ten times) with a pre-rinsed P1000 tip. Afterward, incubate for 5-7 min at 37°C. Add 2.5 volumes of DMEM/F12 to neutralize TrypLE and stop the digestion.
  **Troubleshooting:** see Table 4.
5. Centrifuge at 500 ***g*** for 3 min at room temperature and discard the supernatant completely.
6. Resuspend the pellet with one volume of organoid medium. Hit a P200 tip on a sterile hard surface to decrease the diameter of the pipette tip. Dissociate the organoids by pipetting (40-50 times) with this blunt-end P200 tip until clusters of 10-15 cells are observed microscopically.
7. Add Matrigel to the cell suspension and mix gently to avoid any bubbles. Use a ratio of cells to Matrigel that will allow 10,000 cells in 100 µl of Matrigel.
  **CRITICAL:** To ensure the formation of a firm droplet, do not dilute the Matrigel to a final concentration of less than 3 mg/ml.
8. Dispense 50-100 µl per droplet of the cell-Matrigel suspension into a pre-warmed six-well plate or 5 cm culture dish using a P200 tip.
  **CRITICAL:** Do not place too many droplets into one well or culture dish. If the droplets are too close to each other, they could easily fuse together.
9. Slowly add organoid medium into the well or culture dish (3 ml for six-well plate; 5 ml for 6 cm culture dish).
10. Incubate the plate at 37°C and 5% (v/v) CO_2_. Observe the organoids daily and refresh the medium every 2-3 days.

#### V. (Optional) freezing and thawing of organoids

**Timing: 1-1.5 h**

11. Resuspend the pellet with cell-freezing medium and transfer the mixture into a cryogenic vial. 1 ml freezing medium is sufficient for five droplets of organoids.
12. Place cryogenic vials in a cryogenic freezing container and store at −80°C for 24 h, then transfer the vials to a liquid nitrogen tank for long-term storage.
  **PAUSE POINT:** Cryopreserved organoids may be stored in a liquid nitrogen tank for several years without significantly decreasing organoid-forming efficiency.
13. For thawing of cryopreserved organoids, refer to part A-IV: ‘Thawing of cryopreserved tissue’.

### Part C: co-culture of tumor organoids and CTLs

#### I. Isolation of PBMCs

**Timing: day 1, 1-2 h**

1. Collect patient peripheral blood with 10 ml anticoagulant tubes (e.g. EDTA, heparin and citrate, etc.). Keep the blood at 4°C, and further processing is preferred as soon as possible.
2. Transfer the peripheral blood into a 15 ml centrifuge tube. Dilute the peripheral blood 1:1 with PBS and mix gently.
3. Aliquot Ficoll into a new 15 ml centrifuge tube and then add the diluted peripheral blood slowly along the wall of the tube to the upper layer of Ficoll in a 2.4:3.0 ratio (Ficoll volume:peripheral blood volume).
  **! CAUTION:** Do not disturb the Ficoll layer when adding the peripheral blood. Make sure that the two layers are separated clearly. Alternatively, add the peripheral blood first, then insert the serological pipette intaking Ficoll to the bottom of the 15 ml centrifuge tube, then add Ficoll slowly while drawing out the serological pipette.
4. Centrifuge at 400 ***g*** for 30-40 min at 18-20°C. Set the descending speed as ‘no break’ or ‘10-20% of braking’.
  **CRITICAL:** Mix the Ficoll solution before use by gently inverting the bottle. The above processing is preferred at 18-20°C as temperature is an important factor in stratification.
5. It should be possible to observe four layers after density-gradient centrifugation, corresponding to erythocytes/granulocytes, the Ficoll layer, PBMCs and the plasma/platelet/PBS layer from the bottom to top, respectively.
6. Discard the top plasma/platelet/PBS layer and carefully collect the PBMCs (the white membrane layer) into a new 15 ml tube.
  **CRITICAL:** If there is a large amount of platelet contamination in the PBMC layer, remove the contaminated platelets from PBMCs by secondary centrifuging in a 4-20% gradient sucrose solution or by low-speed centrifugation (60-100 ***g***).
  **Troubleshooting:** see Table 4.
7. Dilute PBMCs with three volumes of PBS and suspend gently. Centrifuge at 400 ***g*** for 10 min and discard the supernatant. Wash PBMCs once more.
8. PBMCs in the tube could be cryopreserved with 10% DMSO+90% FBS or used for the following steps as required.
  **Note:** The cell viability of PBMCs decreases after freezing and thawing, and the average viability is approximately 70-90%.

#### II. Differentiation and maturation of DCs from PBMCs

**Timing: day 3, 30 min; day 6, 30 min**

The collected PBMCs can be divided into two parts, with one-sixth being used to generate DCs, and the remaining PBMCs can be cryopreserved for subsequent isolation of CD8^+^ T cells.

9. Resuspend PBMCs with serum-free RPMI-1640 medium at a density of 3-4×10^6^ cells/ml and seed them in a Petri dish.
10. Incubate the Petri dish at 37°C and 5% (v/v) CO_2_.
11. Wash off the non-adherent PBMCs with PBS gently after 2 h of incubation (the incubation time could be appropriately extended for cell adherence). The adherent cells will be used to generate DCs.
12. Induce DC differentiation from PBMCs with stimulation of GM-CSF (50 ng/ml) and IL-4 (20 ng/ml) in DC differentiation medium (growth factors are listed in Table 2) for 6 days.
13. Change the medium every 3 days by replacing half of the volume of the DC differentiation medium used. Observe morphological changes of DCs during differentiation using a microscope.
  **CRITICAL:** Some adherent cells become semi-suspended or suspended during this process. Keep them and proceed with the maturation process for all the cells.
14. After 6 days of differentiation, induce DC maturation and activation by adding LPS (100 ng/ml) and IFN-γ (500 U/ml) to the DC differentiation medium for 48 h.
  **CRITICAL:** Alternatively, DCs could be also obtained quickly using the DC isolation kit. For tumor-specific stimulation, go to steps 15-18. Confirm DC differentiation and maturation by flow cytometry staining for DC surface co-stimulatory and maturation markers [DC gate: CD1a^+^, CD14^−^, HLA-DRa^+^ and CD11c (ITGAX)^+^; DC maturation: CD40, CD80, CD83 and CD86].

#### III. Generation of tumor specific-CTLs

**Timing: day 8/9, 2-3 h; day 14, 30 min**

15. On the sixth day of DC differentiation, thaw the cryopreserved PBMCs in a 37°C water bath and culture in T cell medium. Two days later, isolate CD8^+^ T cells using the CD8 T cell isolation kit or CD8 nanobeads.
16. At the same time, obtain tumor lysates by six to ten freeze-thaw cycles from the isolated autologous tumor cells, organoids or cell lines. Pulse mature DCs with tumor lysates (200 μg protein/1×10^6^ cells) for 24 h to obtain tumor-antigen-specific DCs.
17. To generate tumor-specific CTLs, incubate antigen-specific DCs with CD8^+^ T cells (collected at step 15) in T cell medium (growth factors are listed in Table 2) in a 5:1 ratio (CD8^+^ T cells: tumor-antigen-specific DCs) for 6 days. CD8^+^ T cells can be isolated from PBMCs or patient tumors using the human CD8^+^ T cell isolation kit.
  **Troubleshooting:** see Table 4.
18. Change the medium every 3 days by replacing half of the volume of T cell medium used.
19. Collect tumor-specific CTLs from the above CD8^+^ T cell and tumor-antigen-pulsed DC mixture using CD8 nanobeads.

#### IV. Co-culture of tumor organoids and CTLs

**Timing: day 14, 1-2 h**

20. Isolate tumor organoids from Matrigel by pipetting up and down in ice-cold PBS. Dissociate part of the organoids into single cells with 0.25% trypsin and count the number of tumor cells per tumor organoid to calculate the number of tumor organoids and CD8^+^ T cells used for co-culture according to the effector:target ratio.
21. Co-culture CTLs and autologous tumor organoids/single cells in a 96-well U-bottom plate with ultra-low attachment surface to perform tumor killing assay with a different effector:target ratio.
22. At the end point of the experiments, collect tumor organoids/cells and CTLs for the tumor cell killing analysis.
  **CRITICAL:** Before co-culturing with CTLs, staining with CellTrace dyes, transfection, gene editing and drug/cytokine treatment could be performed during the culture of tumor organoids, according to the project design. Alternatively, CTLs could be collected from surgical patient tumors in accordance with the corresponding informed consent and the approval of the ethics committee.

## RESULTS AND DISCUSSION

In our hands, the rate of organoid establishment for the normal murine esophageal/tongue epithelium and human GEJ biopsy was almost 100%, whereas the success rate for the formation of organoids derived from human ESCC and BE was 50-60% and 70%, respectively. Human BE and GEJ organoids could be passaged ≥15 times and could be cryopreserved and recovered successfully. Approximately 50-60% of primary ESCC and normal epithelial organoids could be passaged ≥5 times. However, unlike other cancer types, such as breast cancer (Dekkers et al., 2021), we observed a higher growth rate for normal epithelial organoids (split ratio at 1:3-1:5 every 2 weeks) than for ESCC organoids (split ratio at 1:2-1:3 every 2 weeks) for the first five passages in this study.

**Fig. 3.**
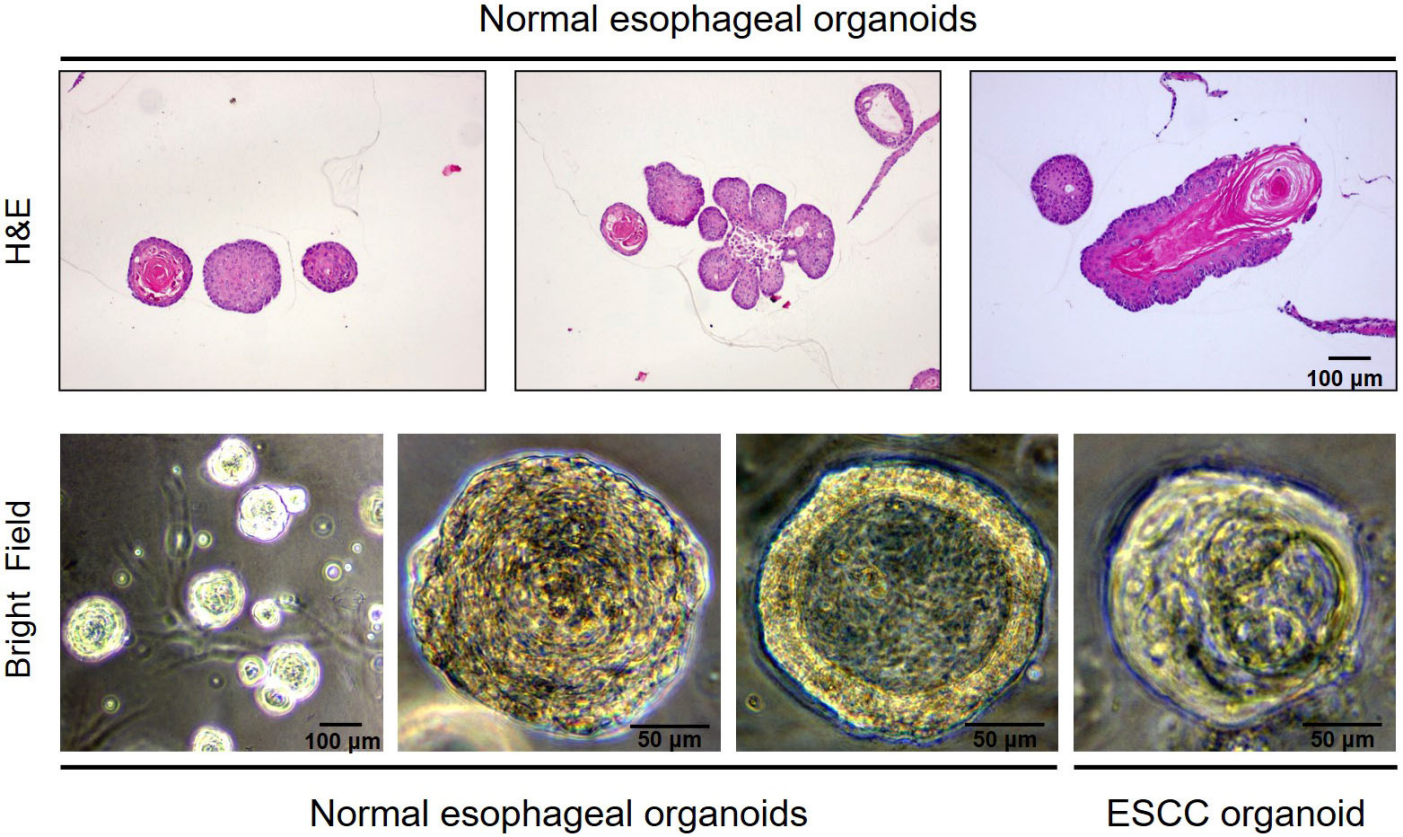
**Characterization of organoids derived from normal esophageal epithelia and ESCC.** Diverse morphological structures of organoids are shown with representative Hematoxylin and Eosin (H&E) staining (upper panel) and bright-field images (on culture day 7) (lower panel). A classical stratified structure, branching structures containing a central lumen, or dense structures can be seen in normal murine esophageal organoids. A human ESCC organoid shows mixed structures (day 14) (lower panel, right). Images are representative of at least three independent experiments. Scale bars: 100 μm (upper panel and the first image of the lower panel); 50 μm (the last three images of the lower panel).

**Fig. 4.**
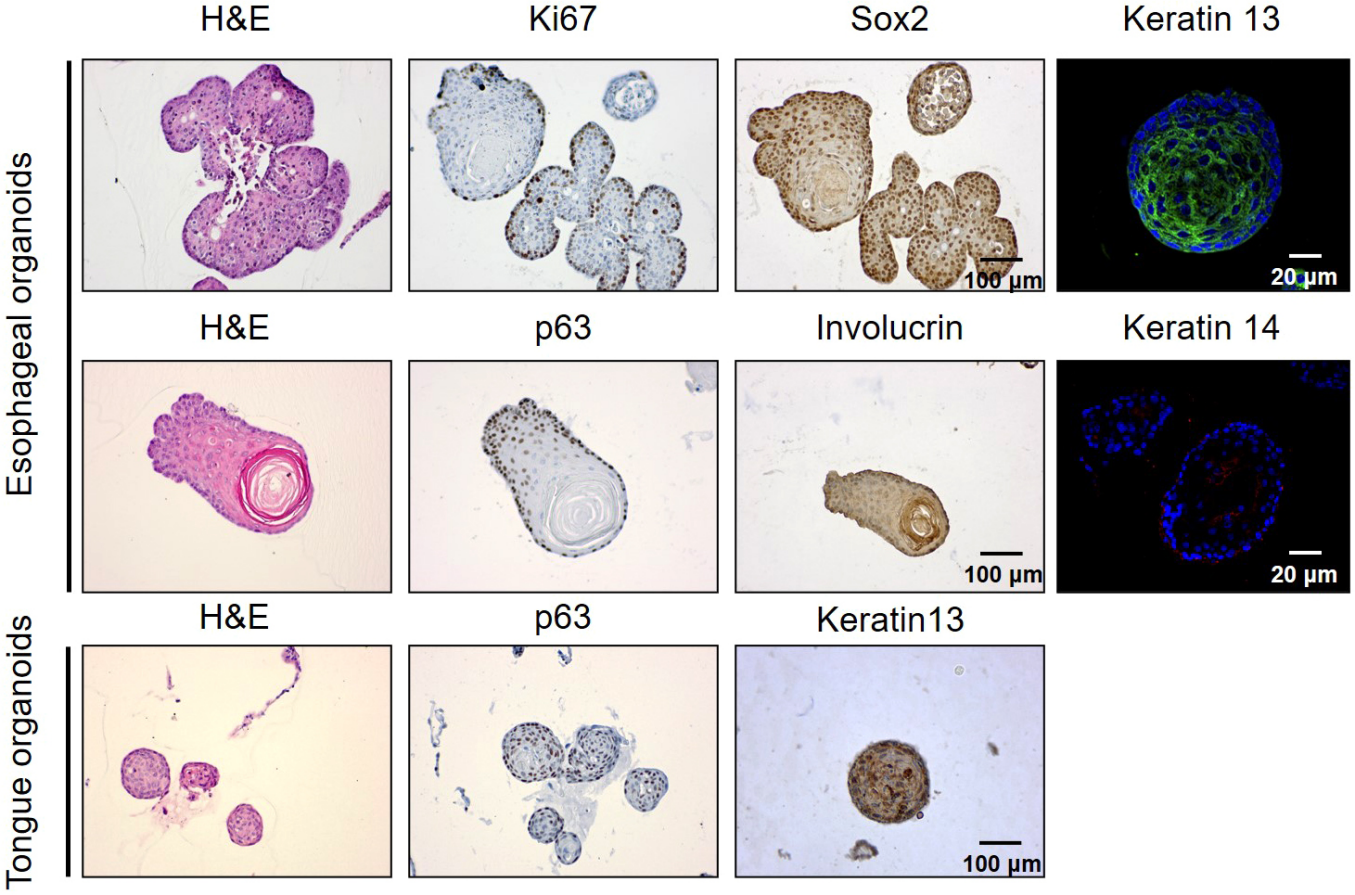
**Characteristics of normal esophageal and tongue organoids.** Representative H&E, immunohistochemistry (IHC) and immunofluorescence images show the morphological characteristics and expression of classical markers at the basal and suprabasal layers of normal esophageal and tongue organoids. Ki67, p63 and Sox2 highly express at the basal cells of organoids. Involucrin (IVL) is an early marker of squamous cell differentiation (center of organoid); keratin 13 (green) and keratin 14 (red) represent differentiated suprabasal and basal layers of organoids, respectively. Images are representative of three independent experiments. Scale bars: 100 μm (H&E and IHC images); 20 μm (IF images).

For the morphology, normal organoids displayed branching structures containing a central lumen, or dense, stratified structures (Fig. 3); whereas most EC tissues showed stratified, cystic (without a central lumen) or mixed structures (Fig. 3). High expression of the epithelial lineage-specific markers p63 (TP63) and Sox2, as well as the proliferative marker Ki67 (MKI67), was observed at the basal cells of normal esophageal and tongue organoids. Expression of keratin 14 (KRT14) and keratin 13 (KRT13) was observed at the basal and differentiated suprabasal cells of normal epithelial organoids, respectively (Fig. 4).

**Fig. 5.**
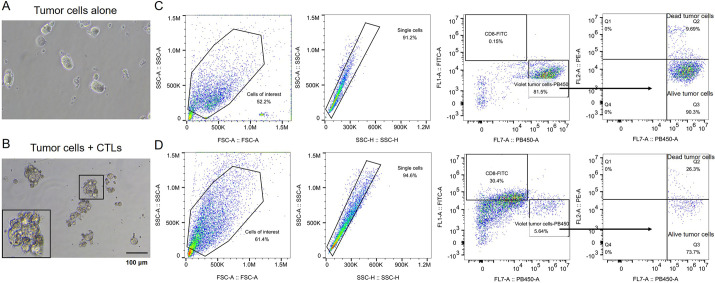
**The tumor organoid killing assay.** (A,B) Bright-field images showing representative tumor cells only (A) and co-cultures of tumor cells and CTLs (B). Tumor cells were dissociated from ESCC organoids. The magnified region (B, bottom left) shows the tumor cell surrounded by activated CD8^+^ T cells (24 h). (C,D) Flow cytometry data analyzing tumor cells after 48 h incubation with (D) or without (C) tumor-antigen-stimulated CD8^+^ T cells. Tumor cells were traced with CellTrace Violet; CD8^+^ T cells were labeled with anti-CD8 antibodies conjugated with FITC fluorochromes. Cell death was measured by propidium iodide uptake (Q2). Live tumor cells are PI^−^ Violet+ cells (Q3). The gating strategy is shown. Images and flow cytometry data are representative of three independent experiments. FSC-A, forward scatter area; SSC-A, side scatter area; SSC-H, side scatter height. FL1, FL2 and FL7 represent different fluorescence channels; FITC, PE and PB450 are CytoFLEX channel names. FL1-A::FITC-A, FITC channel (CD8 staining); FL2-A::PE-A, phycoerythrin channel (propidium iodide); FL7-A::PB450-A, 450/45 BP channel (CellTrace Violet).

Regarding the co-culture *ex vivo* system for studying tumor-immune cell interactions, the key point is the acquisition of CTLs, which can be obtained from either autologous PBMCs or patient tumor-infiltrating CD8^+^ T cells. Herein, we retrieved both CD8^+^ T lymphocytes and DCs from PBMCs, with a portion used for DC differentiation. As shown in Fig. 5A-D, we present an example for the tumor organoid killing assay. Tumor cells were labeled with CellTrace Violet and then co-cultured with CTLs at a 5:1 (effector: target) ratio. After 48 h incubation, all the cells were harvested and analyzed by flow cytometry after anti-CD8-FITC antibody staining and propidium iodide (PI) uptake. The remaining total tumor cells (Violet^+^ cells) and tumor cell death (PI^+^ Violet^+^ cells) were measured, respectively. As expected, CTL cells surrounded tumor cells in the co-culture system (Fig. 5B). Importantly, we observed decreased tumor cell population (Violet^+^ tumor cells), indicating that most tumor cells were killed in the process of co-culture (Fig. 5C,D). Moreover, in the remaining tumor cells, 26.3% were dead (PI^+^ Violet^+^ cells, Q2), and the ratio of alive tumor cells was reduced (73.7%, PI^−^ Violet^+^, Q3) (Fig. 5D). In comparison, the majority of tumor cells were alive (90.3%, Q3) in the tumor-only group (Fig. 5C). These data demonstrate that CD8^+^ T cells stimulated with tumor antigens are able to kill tumor cells in our organoid/CTL co-culture system.

It is difficult to obtain tumor-infiltrating CD8^+^ T cells, which often have low viability *ex vivo*. Contamination of platelets is a common problem during separation of PBMCs from patient peripheral blood with density-gradient centrifugation, possibly caused by altered blood density of the patient with cancer. The contaminated platelets could be partially removed from PBMCs by second centrifugation in 4-20% gradient sucrose solution or by low-speed centrifugation (60-100 ***g***). During the DC differentiation, we observed that both suspended and adherent monocytes could be induced to form mature DCs with successive stimulation by GM-CSF, IL-4, LPS and IFN-γ. For the cytotoxicity assays by co-culturing tumor organoids and tumor-antigen-activated CD8^+^ T cells, strong tumoricidal effects on the autologous tumor organoids are expected. However, tumor cells may escape the killing by activated CD8^+^ T cells due to immunosuppressive mechanisms such as low immunogenicity, antigen presentation deficiency and insufficient chemokine secretion. Therefore, the co-culture *ex vivo* system can be furthered for the identification of strategies to overcome these immune evasion mechanisms. For example, our unpublished work has demonstrated that downregulation of TP63 in ESCC tumor cells strongly promotes tumor killing by CD8^+^ T cells and enhances ICB treatment efficacy.

## Supplementary Material

10.1242/dmm.050319_sup1 Supplementary information
