# Supplementary material for: Establishing mouse and human oral esophageal organoids to investigate the tumor immune response
Source: Dis Model Mech. 2024 Jan 23;17(1):dmm050319. doi: 10.1242/dmm.050319 (PMC10846528; doi:10.1242/dmm.050319)
Supplement: Supplementary information [file dmm-17-050319-s1.pdf]

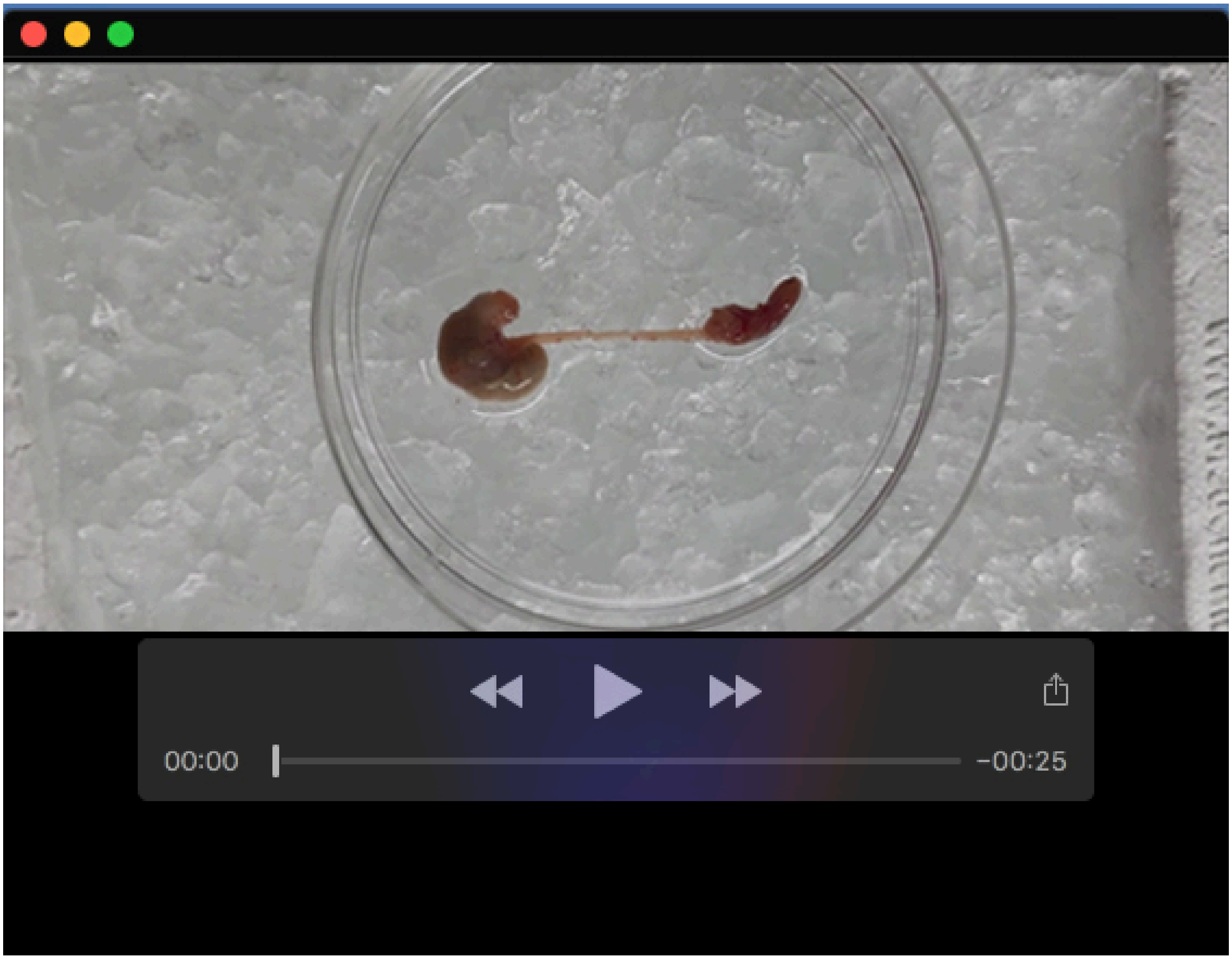

Movie 1. Isolation of murine esophageal epithelium.

Disease Models & Mechanisms • Supplementary information

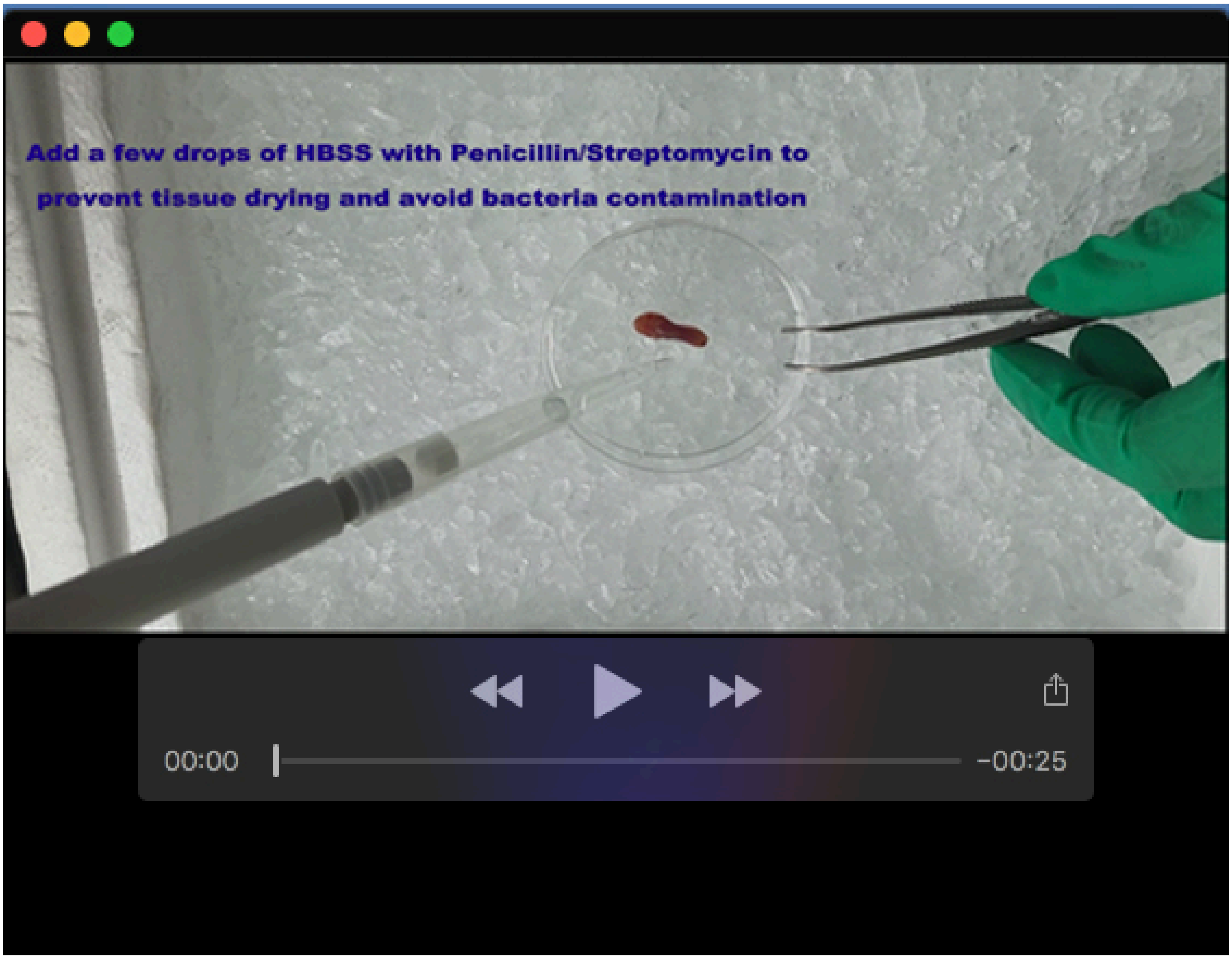

Movie 2. Isolation of murine tongue epithelium.
